# Clinicopathologic features of KRAS G12C-mutated non-small cell lung carcinomas:insights from 279 retrospective cases

**DOI:** 10.1007/s00428-026-04526-x

**Published:** 2026-04-15

**Authors:** Martina Bradová, Petr Slavík, Tomáš Vaněček, Petr Martínek, Petr Grossmann, Stanislav Kormunda, Kristýna Behenská, Martin Svatoň, Miloš Pešek, Tomáš Jirásek, Zuzana Špůrková, Petra Hroudová, Hana Mrázková, Barbora Hořavová, Jaromír Roubec, Martin Baník, Petr Mukenšnábl, Michal Michal, Marián Švajdler

**Affiliations:** 1 Charles University, Faculty Hospital Plzen, Department of Pathology, Faculty of Medicine in Plzen, Czech Republic, 1Charles University, University Hospital Plzen, Plzen, Czech Republic; 2 Bioptic Laboratory, Ltd, Plzen, Czech Republic; 3 Department of Pneumology and Phthisiology, Faculty of Medicine in Pilsen, Charles University in Prague, Prague, Czech Republic; 4 Department of Pathology, Liberec Regional Hospital, Liberec, Czech Republic; 5 Department of Clinical and Molecular Pathology, Faculty of Medicine and Dentistry, Palacký University, Olomouc, Czech Republic; 6 Department of Pathological Anatomy, Bulovka University Hospital, Bulovka, Czech Republic; 7 Department of Pathology, Hospital Ceske Budejovice, a.s., Ceske Budejovice, Czech Republic; 8 Regional Health Hospital of the Ústí Region, a.s. - Most Hospital, branch, Ústí, Czech Republic; 9 Department of Pathology, Frýdek-Místek Hospital, Frýdek-Místek , Czech Republic; 10 AGEL Ostrava-Vítkovice Hospital Inc., Ostrava-Vítkovice, Czech Republic; 11 Department of Pathology, Karlovy Vary Hospital, Karlovy Vary , Czech Republic; 12 Sikl’s Department of Pathology, Faculty of Medicine in Pilsen, Charles University, E. Benese 13, Pilsen, 305 99 Czech Republic

**Keywords:** Lung, KRAS G12C, NSCLC, Adenocarcinoma, Molecular genetics, Statistics

## Abstract

KRAS G12C-mutated non-small cell lung carcinoma (NSCLC), caused by a glycine-to-cysteine substitution at codon 12, is associated with poor prognosis and is now targetable with specific inhibitors. We retrospectively analyzed 279 KRAS G12C-mutated NSCLC cases (2017–2023) from our registry with available histologic, immunohistochemical, and molecular data. The cohort included 279 patients (125 females, 151 males; mean age 67 years, range 29–91). Most tumors were primary lung carcinomas (n = 229, 82%), while 45 (16%) were metastatic at presentation. Morphologic evaluation was available in 240 tumors: 37% showed solid squamous cell carcinoma (SCC)-like features, 61% rhabdoid/plasmacytoid morphology, and 17% sarcomatoid features. Adenocarcinoma-associated patterns were present in 67 cases, often mixed, and focal solid growth occurred in 77%. TTF1, Napsin A, and CK7 were positive in 86%, 87%, and 98%, respectively, whereas squamous markers were infrequent (p40/p63 7%, CK5/6 8%). PD-L1 expression was detected in 65%. Co-mutations most commonly involved *TP53* (n = 27) and *STK11* (n = 12); *IDH1*/*2, PIK3CA*, and *CTNNB1* mutations occurred in four cases each, *MET* in two cases, and *BRAF*, *FGFR2*, *FGFR3*, and *GNAS* in one case each. Two gene fusions were identified (*LRP12*::*NRG1*, *FGFR3*::*TACC3*). Mean survival was 1.89 years, with one- and five-year survival rates of 54% and 25%. KRAS G12C-mutated NSCLC is clinically aggressive and frequently shows solid growth with rhabdoid, plasmacytoid, or SCC-like morphology, which may lead to misclassification and missed genetic testing. Immunohistochemistry and molecular profiling are essential for accurate classification and enabling targeted therapy.

**Supplementary Information:**

The online version contains supplementary material available at 10.1007/s00428-026-04526-x.

## Introduction

Non-small cell lung carcinoma (NSCLC) accounts for approximately 80–85% of lung cancers and is characterized by a high frequency of oncogenic driver mutations that increasingly guide therapeutic decision-making [1–4]. Among these, KRAS mutations represent the most prevalent molecular alterations in NSCLC, while treatment has evolved dramatically with the advent of molecular profiling, enabling the implementation of targeted therapies, including tyrosine kinase inhibitors, as well as immune checkpoint inhibitors (ICIs) [4].

The *RAS* gene family encodes membrane-bound GTP-binding proteins (GTPases) that regulate cell signaling [5, 6]. It comprises three isoforms (*HRAS*, *NRAS*, and *KRAS*), which are among the most frequently mutated genes in human cancers, particularly lung, colon, and pancreatic [7, 8].

*KRAS* is the most commonly altered member, with missense mutations typically at codons 12, 13, or 61 [8]. G12 substitutions are the most prevalent in NSCLC and pancreatic ductal adenocarcinoma, but specific variants differ by tumor type: G12C (glycine → cysteine) is most frequent in NSCLC yet rare in other cancers [8–10].

RAS-targeting was long deemed undruggable due to KRAS's exclusive GTP binding and high affinity even at low GTP levels [5, 6]. On May 28, 2021, Food and Drug Administration (FDA) approved sotorasib (AMG-210) [11] followed by adagrasib (MRTX849) on December 12, 2022 [12] as KRAS G12C inhibitors for adults with locally advanced or metastatic NSCLC.

In the present study, we retrospectively analyzed clinicopathologic features of 279 KRAS G12C-mutated NSCLCs.

## Materials and methods

### Histology and immunohistochemistry

A retrospective search of the authors' molecular-genetic registry was conducted. Among 2,348 NSCLC cases analyzed by next-generation sequencing between 2017 and 2023, 279 primary or metastatic tumors harboring *KRAS* G12C mutation were identified. Of the 2,348 cases, 1,823 were tested using RNA, 492 using both DNA and RNA, and 33 using DNA only. Among the 279 *KRAS* G12C-mutated cases, 214 were tested using RNA, 60 using both DNA and RNA, and 5 using DNA only.

Histopathologic and immunohistochemical features were reviewed. The study complied with the Ethics Committee guidelines of the Faculty Hospital in Pilsen; informed consent was not required. Follow-up data were collected from referring pathologists, clinicians, hospital records, and insurance reports.

For conventional microscopy, tissues were formalin-fixed, paraffin-embeded (FFPE), sectioned, and stained with hematoxylin and eosin.

For immunohistochemistry, 4-μm-thick sections were cut from paraffin blocks and mounted on positively charged slides (TOMO, Matsunami Glass IND, Osaka, Japan). Sections were processed on a BenchMark ULTRA (Ventana Medical Systems, Tucson, AZ), deparaffinized and subjected to heat-induced epitope retrieval by immersion in a CC1 solution (pH 8.6) at 95 °C and CC2 solution (pH 6.0) at 92 °C. All primary antibodies used in this study are summarized in (Table 1). Visualization was performed using the ultraView Universal DAB Detection Kit (Roche, Tucson, AZ) or ultraView Universal Alkaline Phosphatase Red Detection Kit (Roche, Tucson, AZ). The slides were counterstained with Mayer’s hematoxylin. Appropriate positive and negative controls were employed.

**Table 1:** Antibodies Used for Immunohistochemical Study

Antibody Specificity	Clone	Dilution	Antigen Retrieval/time	*Source*
SMARCA4	EPNCIR111A	1:1000	CC1/52 min	*Abcam*
SMARCB1	MRQ-27	RTU	CC1/52 min	*Ventana*
AE1/3	AE1/AE3 + PCK26	RTU	CC1/20 min	*Ventana*
OSCAR	IsoType:IgG2a	1:100	EnVision High pH/30 min	*Covance*
CAM5.2	CAM5.2	RTU	CC1/36 min	*Ventana*
Claudin 4	3E2C1	1:200	CC1/52 min	*Thermo Scientific – Life Technologies*
MOC-31	MOC-31	1:50	CC1/52 min	*Abcam*
CK5/6	D5/16B4	1:50	EnVision High pH/30 min	*Dako*
CK7	OV-TL 12/30	RTU	EnVision High pH/30 min	*Dako*
p63	DAK-p63	RTU	EnVision Low pH/30 min	*Dako*
p40	BC28	RTU	CC1/52 min	*Biocare Medical*
TTF1				
PD-L1	22C3	1:50	EnVision Low pH/30 min	*Dako*
ALK	OTI1A4	RTU	EnVision Low pH/30 min	*Dako*
*ROS1*	*D4D6®*	*1:50*	*CC1/64 min*	*Cell Signaling*

RTU, ready to use; CC1, EDTA buffer pH 8.6 at 95 °C; CC2, citrate buffer pH 6.0 at 92 °C; EnVision High pH 9.0 at 97 °C; EnVision Low pH 6.0 at 97 °C; min, minutes

Predictive biomarker testing was performed using established protocols. PD-L1 expression was assessed by Tumor Proportion Score (TPS) calculated as the percentage of PD-L1-positive tumor cells among all viable tumor cells. Cut offs were defined negative (< 1%), low (1–49%) and high (≥ 50%). ROS1 expression was evaluated using rabbit monoclonal antibody (D4D6 clone, Cell Signaling Technology, Danvers, MA, USA) and scored semi-quantitatively based on staining intensity and percentage of positive tumor: 0 (negative) – no staining or faint staining in < 10% of tumor cells; 1 + (weak) – faint, barely perceptible staining in ≥ 10% of tumor cells; 2 + (moderate) – distinct cytoplasmic staining in ≥ 10% of tumor cells; 3 + (strong) – intense granular cytoplasmic staining in ≥ 10% of tumor cells. [13, 14]. Tumors which scores 1 +, 2 +, or 3 + were confirmed by FISH or NGS, while negative cases were not tested further.

ALK expression was assessed using the OTI1A4 clone (OriGene Technologies, Rockville, MD, USA) [15] interpreted using a binary scoring system: positive when strong granular cytoplasmic staining was present in ≥ 10% of tumor cells, and negative when staining was absent or limited to background levels. Equivocal or discordant cases were confirmed by FISH or NGS.

### Molecular genetic testing

#### Next generation sequencing (NGS)

Depending on the sample size, up to 3 FFPE Sects. (10 µm thick) were sectioned and DNA and RNA was extracted using DNA/RNA FFPE or DNA/RNA FFPE RSC kits (automated on Maxwell RSC 48 Instrument, Promega, Madison, Wisconsin, USA) according to the manufacturer's instructions. DNA and RNA was quantified using the Qubit Broad Range DNA/RNA Assay Kits (ThermoFisher Scientific) and input for each sample’s library preparation was set to 250 ng RNA or 100 ng DNA for fusion or SNV detection respectively. The PreSeq RNA QC Assay using iTaq Universal SYBR Green Supermix (Biorad, Hercules, CA) was performed to assess RNA quality. Samples with the cycle threshold value smaller than 30 continued with target enrichment.

The FusionPlex CTL kit containing 195 targets in 36 genes or FusionPlex Lung v2 with 323 gene specific primers targeting 17 genes mutated in non-small cell lung cancer were used for fusion and SNV detection from RNA. VariantPlex CTL kit was used for DNA SNV analysis in 290 regions of interest across 31 genes. Library preparations were performed following Fusion Plex or Variant Plex Protocols for Illumina (ArcherDX Inc., Boulder, CO) and were quantified following the Library Quantification for Illumina Libraries protocol (KAPA, Wilmington, MA) assuming a 300 or 350 bp fragment lengths respectively. Samples were multiplexed and sequenced on a NextSeq 550 or Novaseq 6000 sequencers (Illumina, San Diego, CA) spiked with up to 20% PhiX to ensure library nucleotide diversity. The multiplexing was calculated to obtain a minimum of 3 milion read pairs for FusionPlex CTL and VariantPlex CTL or 2 milion read pairs for FusionPlex Lung V2. The analysis of the sequencing results was performed using the Archer Analysis software (version 5–7; ArcherDX Inc.). Parameters for filtering strong fusions as determined by the Archer Analysis software were set to a minimum of 5 fusion supporting reads with a minimum of 3 unique start sites. For SNV and indel detection the parameters were set to 5% allelic frequency, minimum depth 40x, alternate observations > 5, unique start sites of alternate observation > 3, less than 0,5% population frequency using GnomAD database and non-synonymous variant consequence. All filtered variant ‘s raw reads were visualy inspected by two molecular biologists to exclude potential artefacts and confirmed variants were reported.

#### Detection of ALK and ROS1 gene rearrangements by FISH

Prior to performing FISH, hematoxylin and eosin-stained slides were examined to determine the areas for cell counting. Then, a 4-µm-thick formalin-fixed, paraffin-embedded section was placed onto a positively charged slide. The unstained slide was routinely deparaffinized and incubated in the 1 × Target Retrieval Solution Citrate pH 6 (Dako, Glostrup, Denmark) for 40 min at 95 °C, subsequently cooled for 20 min at room temperature in the same solution and washed in deionized water for 5 min. The slide was digested in protease solution with pepsin (0.5 mg/mL) (Sigma Aldrich, St Louis, MO, USA) in 0.01 M HCl at 37 °C from 45 to 60 min according to the sample conditions. The slide was then rinsed in deionized water for 5 min, dehydrated in a series of ethanol solutions (70%, 85%, 96% for 2 min each), and air-dried.

For the detection of the break of *ALK* resp. *ROS1* genes were used commercial probes Human ALK Gene Fusion Detection Probe Detection Kit resp. ROS1 (6q22) Gene Fusion Detection Probe Detection Kit (Wuhan HealthCare Biotechnology Co., Ltd., Wuhan City, China).

An appropriate amount of both factory premixed probes was applied on the specimens, covered with a glass cover slip, and sealed with rubber cement. The slide was incubated in the ThermoBrite instrument (StatSpin/Iris Sample Processing, Westwood, MA, USA) with codenaturation parameters of 85 °C for 8 min and hybridization parameters of 37 °C for 16 h. The rubber-cemented cover slip was then removed, and the slide was placed in posthybridization wash solution (2 × SSC/0.3% NP-40) at 72 °C for 2 min. The slides were air-dried in the dark, counterstained with DAPI II (Vysis/Abbott Laboratories, Abbott Park, IL, USA), covered with slip, and immediately examined under an Olympus BX51 fluorescence microscope using a × 100 objective and filter sets Triple Band Pass (DAPI/Spectrum Green/Spectrum Orange), Dual Band Pass (FITC/Spectrum Orange), and Single Band Pass (Spectrum Green or Spectrum Orange).

A minimum of 50 randomly selected nonoverlapping interphase tumor cell nuclei were evaluated for the presence of a specific positive break pattern where the samples were considered positive when ≥ 15% of nuclei show break signals as previously described by McCoach et al. [16].

## Statistical analysis

All analyses were performed using SAS software (SAS Institute Inc., Cary, NC, USA).

Descriptive statistics were calculated for continuous variables (mean, standard deviation, variance, median, interquartile range, minimum, and maximum) and categorical variables (absolute and relative frequencies). Selected parameters were visualized using box plots, histograms, and pie charts. Groups comparisons were conducted using nonparametric tests (Wilcoxon two-sample test), and correlations were assessed with Spearman’s coefficient. Overall survival analysis was performed using Kaplan–Meier survival curves. The effects of individual factors were tested using the Log-rank test, Gehan–Wilcoxon test, and Cox proportional hazards regression model. Optimal cut-offs and risk groups were identified using Cox regression by maximizing the test statistic. The clinical impact of individual factors was expressed as a Hazard Ratio. Several multivariate models were constructed. Independent predictors were identified using stepwise Cox regression analysis. The multivariate analysis was further developed using a multivariate Cox regression model presented in the form of Classification and Regression Trees. The statistical significance of the regression tree branches identified by the Cox regression model was verified using the Log-rank test.

Statistical significance was set at α = 0.05.

## Results

The cohort comprised 151 men and 125 women (sex unknown in 3 cases), aged from 29 to 91 years (mean age 66.9; median 68). Specimens included 37 surgical resections (13%), 58 excisional biopsies (21%), 150 needle biopsies (54%), and 28 cytology samples (10%). In six cases (2%), the type of procedure was unknown. Cytology specimens were insufficient for assessment some histological parameters and most immunohistochemical tests.

Histologic classification followed WHO criteria (World Health Organization Classification: Thoracic tumours) [17], and cytology was categorized using WHO Reporting System for Lung Cytopathology [18].

Clinical, histologic, immunohistochemical, molecular findings, staging, and follow-up are summarized in Table 2.

**Table 2:** Clinical features, histological parameters, immunohistochemical, molecular genetic findings and staging of the disease

Clinical features	No. of cases (279)	
Sex Male/Female	151/125 (in 3 cases sex not known)	
Mean age (range)	66.9 years (29–91)	
Diagnosis	**No. of cases (279)**	**(%)**
Invasive non-mucinous adenocarcinoma and/or NSCLC most likely adenocarcinoma	233	84
NSCLC NOS	21	8
Invasive mucinous adenocarcinoma	12	4
Mixed invasive mucinous and non-mucinous adenocarcinoma	8	3
Non-keratinizing squamous cell carcinoma	1	0.4
Adenosquamous carcinoma	1	0.4
Not known	3	1
Smoking history	**No. of cases (230)**	**(%)**
• Smoker	151	66
• Ex-smoker	74	32
• Non-smoker	5	2
Localization of the specimen	**No. of cases (279)**	**(%)**
Lung/bronchus	229	82
Locoregional spread/metastasis		
• Pleura/pleural cavity/chest wall	18	6
• Hilar/mediastinal lymph nodes	9	3
Distant metastasis		
• Neck and axillary lymph nodes	5	2
• Skin	5	2
• Brain/cerebellum	5	2
• Abdominal cavity	1	0.4
• Bones	2	0.7
• Unknown location	5	2
Type of growth	**No. of cases (240)**	**(%)**
• Solid	184	77
• Cribriform	25	10
• Lepidic	34	14
• Acinar	19	8
• Papillary, micropapillary	37	15
• Other glandular growth patterns	107	45
• Not assessed	39	16
Other histological features	**No. of cases (158)**	**(%)**
• Desmoplasia	120	76
• Necrosis	48	30
• Inflammation	33	21
Cellular features	**No. of cases (240)**	**(%)**
• SCC-like	89	37
• Rhabdoid/plasmacytoid/epithelioid	146	61
• Sarcomatoid	40	17
• Small cells	3	1
• Adenocarcinoma (cribriform or serrated)	67	28
• Cannot be assessed	6	3
Immunohistochemistry	**No. of positive cases/No. of tested cases**	**(%)**
• TTF1	210/244	86
• Napsin A	169/195	87
• CK7	87/89	98
• p40/p63	11/162	7
• CK5/6	7/83	8
• PD-L1		
• 1–49%	51/233	22
• ≥ 50%	100/233	43
• negative	82/233	35
• ALK1		
• positive	22/204	11
• negative	182/204	89
• ROS1		
• positive	15/179	8
• negative	164/179	92
Molecular genetics of *KRAS G12C* mutated NSCLC	**No. of cases (280)**	**(%)**
Gene mutations		
• *TP53*	27	9
• *STK11*	12	4
• *CTNNB1*	4	1
• *IDH1/2*	4	1
• *PIK3CA*	4	1
• *MET*	2	1
• Other (*BRAF, FGFR2, FGFR3, GNAS*)	4	1,4
Gene fusions		
• *LRP12::NRG1; FGFR3::TACC3*	2	0.7
Follow-up	**No. of cases (257)**	**(%)**
• DOD	134	52
• AWD	42	16
• ANED	19	7
• DOUR	11	4
• LOF	51	20
• NA	22	9
Stage	**No. of cases (231)**	**(%)**
• IA	22	10
• IB	11	5
• IIA	3	1
• IIB	14	6
• IIIA	25	11
• IIIB	22	10
• IIIC	4	2
• IVA	79	34
• IVB	51	22

*ANED* alive not evidence of disease, *AWD* alive with disease, *DOD* died on the disease, *DOUR* died on unrelated reasons, *LOF* lost to follow-up, *NA* not available, *NSCLC* non-small cell lung carcinoma, *SCC* squamous cell carcinoma

Final diagnoses included invasive non-mucinous adenocarcinoma or NSCLC-most likely adenocarcinoma in 233 cases (84%), NSCLC not otherwise specified (NOS) in 21 (7.5%), invasive mucinous adenocarcinoma in 12 cases (4.3%), mixed mucinous/non-mucinous adenocarcinomas in 8 (3%), non-keratinizing SCC in 1 (0.4%), and adenosquamous carcinoma in 1 (0.4%). In three cases (1%), only molecular data confirming KRAS G12 mutated (adeno)carcinoma were available; the exact tumor location, biopsy procedure, histology, and immunoprofile were unknown.

### Histomorphological features

**Fig. 1:**
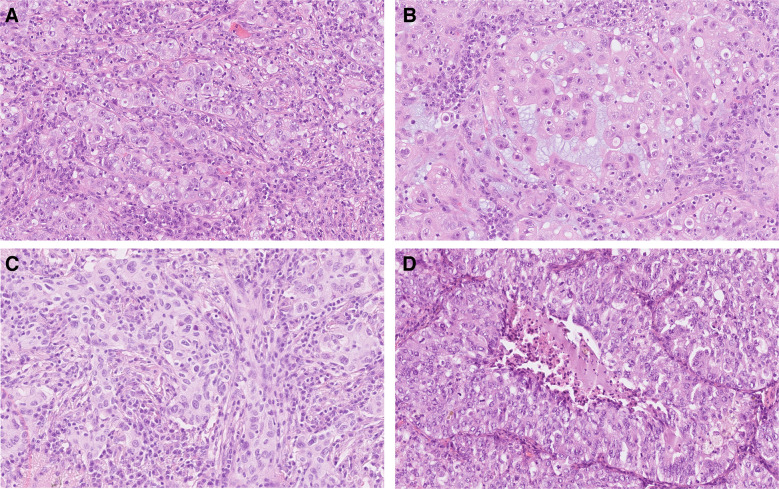
Over 70% of cases were of solid and nested morphology with single cell pattern or single cell lines (**A**). Some cases developed acantholytic areas throughout tumor content (**B**). Nested morphology and high nuclear and cellular pleomorphism was predominant in the cohort (**C**), some showing comedo-like necrosis or large necrotic areas (**D**)

**Fig. 2:**
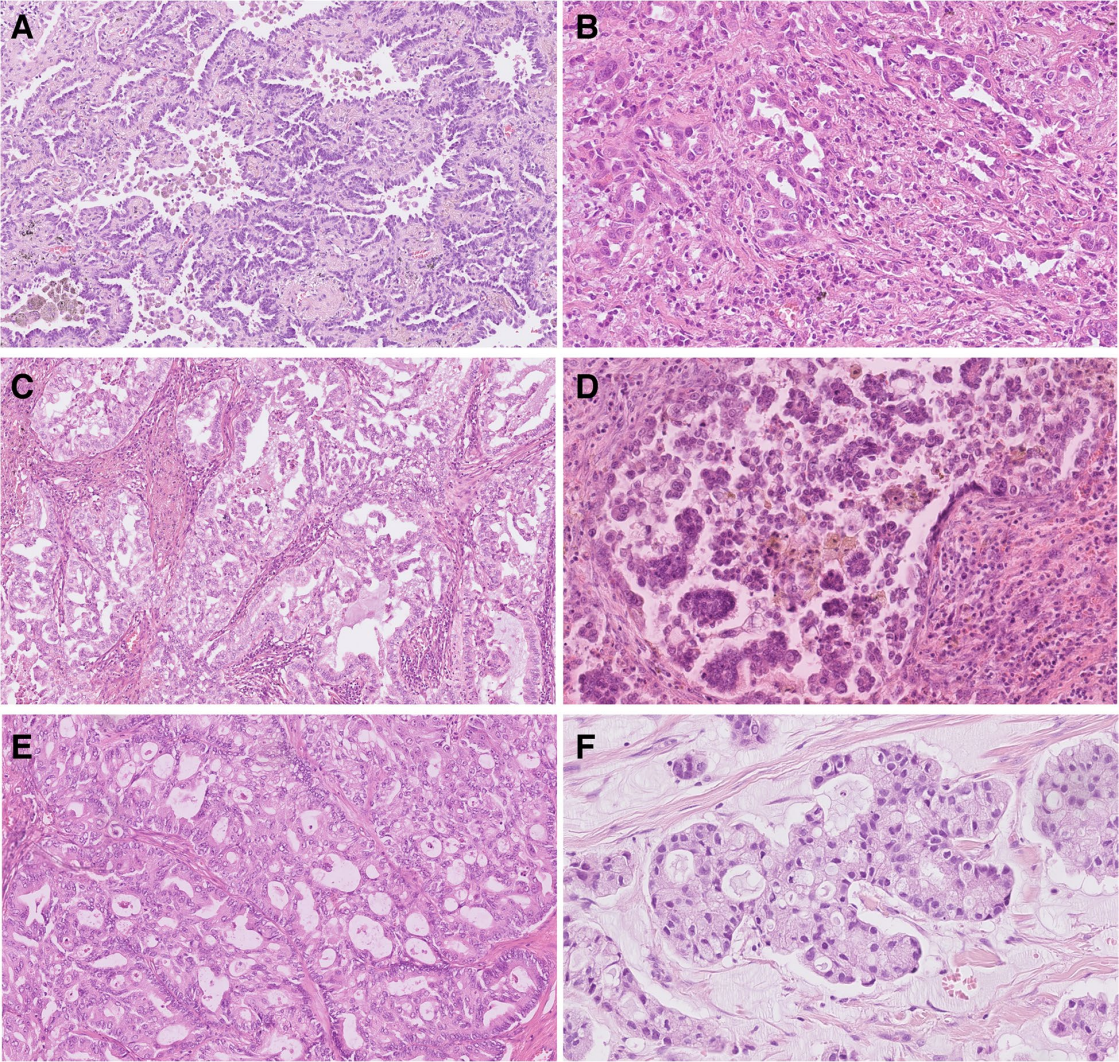
Adenocarcinoma morphology was less obvious and if present than it comprised at most 50% of the tumor volume there were different growth patterns including lepidic (**A**), acinar (**B**), pseudoglandular with papillary architecture (**C**), micropapillary pattern (**D**), cribriform architecture **(E)**, and mucinous adenocarcinoma morphology (**F**)

**Fig. 3:**
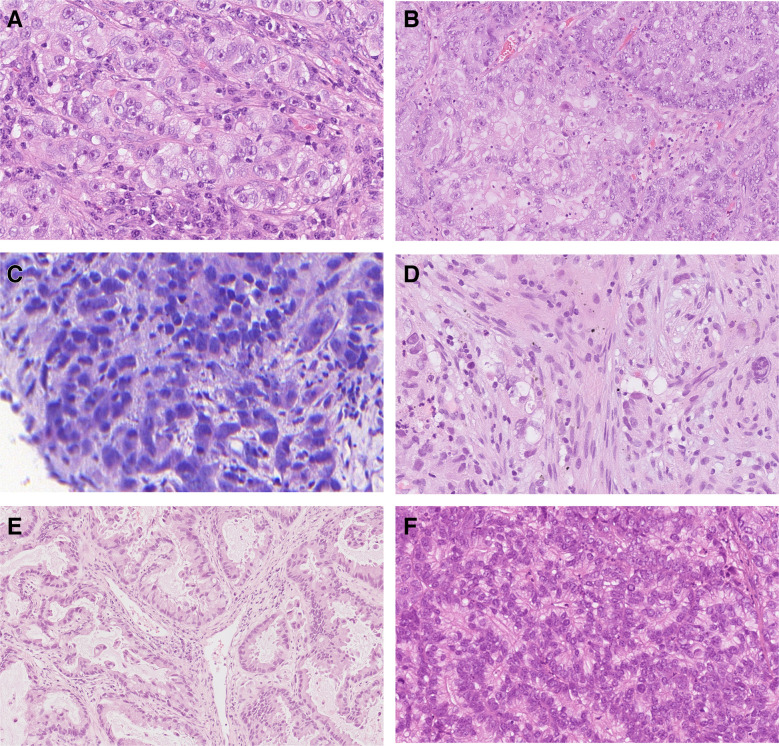
Tumor cell morphology was epithelioid (rhabdoid) (**A**) with squamous-like morphology **(B)**. Basaloid and small cell morphology was present in 3 cases **(C)**. Spindle cell to sarcomatoid appearance was recorded in 17% **(D)**. Cases with adenocarcinoma morphology exhibited features reminiscent in some cases of serrated adenocarcinomas of the intestine **(E)** and, prostatic adenocarcinomas with cribriform architecture **(F)**

Architecture was assessable in 240 cases (86%) **(**Figs. 1, 2 and 3**).** Solid growth predominated, present in 184 cases (77%) and accounting for ≥ 50% of tumor volume when observed **(**Fig. 1A–D). Acantholysis was occasionally present in SCC-like areas **(**Fig. 2B**).** Necrosis was common, appearing as comedo-like or extensive tumor necrosis **(**Fig. 1D**).** Adenocarcinoma-like patterns were less common, typically involving ≤ 50% of tumor volume. Lepidic (*n* = 34, 14%), acinar (*n* = 19, 8%), papillary and micropapillary (*n* = 37, 15%), cribriform (*n* = 25, 10%) and other glandular (*n* = 107, 77%) growth patterns occurred in various combinations, often with solid growth **(**Fig. 2A–F**).**

Tumor cell morphology was epithelioid to poorly differentiated/rhabdoid in 61% (*n* = 146) **(**Fig. 1A-D, 2A, 2B**),** with SCC-like features in 37% (*n* = 89) (including two cases with true squamous differentiation diagnosed as non-keratinizing SCC adenosquamous carcinoma) **(**Fig. 3A**)**, small cell/basaloid in 1% (*n* = 3) **(**Fig. 3C**)**, and sarcomatoid/spindle-cell in 17% (*n* = 40) **(**Fig. 3D**)**.

Hobnail, clear-cell, serrated-like, and intestinal/penicillate features were present in 184 cases (86%) **(**Fig. 2A–F, 3E, 3F**)**; in half of these cases the pattern was very focal (< 10%), while in the other half it comprised at most 50% of the tumor volume. Most tumors contained multiple cell phenotypes within a single lesion.

### Immunohistochemical findings

Immunohistochemical results adapted to tumor histological or cytological diagnosis are summarized in Table 3**.** Cases diagnosed as invasive non-mucinous adenocarcinoma or NSCLC most likely adenocarcinoma, were positive for TTF1 in 192 cases (82%) and positive for Napsin A in 158 (68%). CK7 was expressed in 71 cases (30%), and was expressed in all tested TTF1/Napsin A-negative tumors (*n* = 11) except one triple-negative case. CK5/6 and p40/p63 were rarely positive (2% and 3%), usually alongside TTF1. NSCLC NOS group consisting of 21 cases showed expression of TTF1 in five cases, Napsin A in two cases and CK7 in seven cases, while CK5/6 and p40/p63 were expressed in two and one case respectively. Invasive mucinous adenocarcinoma (12 tested cases) expressed TTF1 in nine cases, Napsin A in six cases, and CK7 in seven. CK5/6 and p40/p63 were negative in all tested cases (2 and 4 cases, respectively). From eight tested mixed invasive mucinous/non-mucinous adenocarcinomas cases, TTF1 was positive in five, Napsin A in three, and CK7 in one; p40/p63 was negative in all three tested cases. Non-keratinizing squamous cell carcinoma (NKSCC) and adenosquamous carcinoma (ASC) (1 case each) were negative for TTF1 and Napsin A. NKSCC was positive for p63/p40 and CK5/6; ASC expressed CK7 and p40/p63 but was CK5/6-negative.

**Table 3:** Immunohistochemical findings related to the histological or cytological tumor diagnosis

Diagnosis		Invasive non-mucinous adenocarcinoma or NSCLC most likely adenocarcinoma (*N* = 234)	NSCLC not otherwise specified (*N* = 21)	Invasive mucinous adenocarcinoma (*N* = 12)	Mixed invasive mucinous and non-mucinous adenocarcinomas (*N* = 8);	Non-keratinizing squamous cell carcinoma (*N* = 1);	Adenosquamous carcinoma (*N* = 1)
Antibody	Expression						
TTF1	positive	192	5	9	5	0	0
	negative	19	9	2	1	1	1
	not done	23	7	1	2	0	0
Napsin A	positive	158	2	6	3	0	1
	negative	16	6	2	1	1	0
	not done	60	13	4	4	0	0
CK7	positive	71	7	7	1	0	1
	negative	1	1	0	0	0	0
	not done	162	13	5	7	1	0
CK5/6	positive	5	1	0	0	1	0
	negative	73	2	2	0	0	0
	not done	156	18	10	8	0	1
p40/p63	positive	7	2	0	0	1	1
	negative	135	10	4	3	0	0
	not done	92	9	8	5	0	0
PD-L1	1–49%	43	3	3	3	0	0
	≥ 50%	89	7	1	2	0	1
	negative	70	4	6	1	0	0
	not done	32	7	2	2	1	0
ALK	positive	20	1	1	0	0	0
	negative	156	13	7	6	0	1
	not done	58	7	4	2	1	0
ROS1	positive	13	0	1	1	0	0
	negative	141	13	6	4	0	1
	not done	80	8	5	3	1	0

*NSCLC* non-small cell lung carcinoma

### Predictive biomarkers expression

PD-L1 was evaluated in 234 cases; 46 cases were not tested due to insufficient tissue or technical issues. Among the tested cases, PD-L1 was positive in 1–49% of tumor cells in 52 cases (22.2%), in ≥ 50% of tumor cells in 100 cases (42.7%), and negative in 82 cases (35.0%). The mean PD-L1 expression was 39%.

ALK was evaluated in 205 cases; 22 cases (10.7%) were positive and 183 (89.3%) negative. ROS1 was tested in 180 cases; 15 (8.3%) were positive and 165 (91.7%) negative. However, molecular genetic testing did not confirm any underlying genetic alterations.

### Molecular genetic findings

In addition to the *KRAS* G12C mutation detected in all cases, additional mutations were found in 58 cases (20.8%), most commonly *TP53* (*n* = 27) and *STK11* (*n* = 12), followed by *IDH1/2* (*n* = 4), *PIK3CA* (*n* = 4), *CTNNB1* (*n* = 4), *MET* (*n* = 3), and single cases of *BRAF, FGFR2, FGFR3,* and *GNAS*.

Two gene fusions were identified: *LRP12::NRG1,* and *FGFR3::TACC3*.

### Follow-up and survival outcomes

At diagnosis, stage distribution was: stage IA 22 patients (10%), stage IB 11 (5%), stage IIA 3 (1%), stage IIB 14 (6%), stage IIIA 25 (11%), stage IIIB 22 (10%), stage IIIC 4 (2%), stage IVA 79 (34%), and stage IVB 51 (22%).

Follow-up was available in 257 cases (92%). Nineteen patients were alive without evidence of disease (mean 31.7 months; range 1–54; median 32), 42 alive with disease (mean 35.6 months; range 2–102; median 28), 134 died of disease (mean 9.8 months; range 0–72; median 4), 11 died of unrelated causes (mean 14.2 months; range 1–36; median, 12), and 51 were lost to follow-up (mean 7.7 months; range, 1–36; median, 2). Treatment data were available in 247 patients, who received either single or combined modalities, including chemotherapy in 111 cases (45%), radiotherapy in 82 (34%), immunotherapy in 56 (23%), and surgery in 49 (20%).

**Fig. 4:**
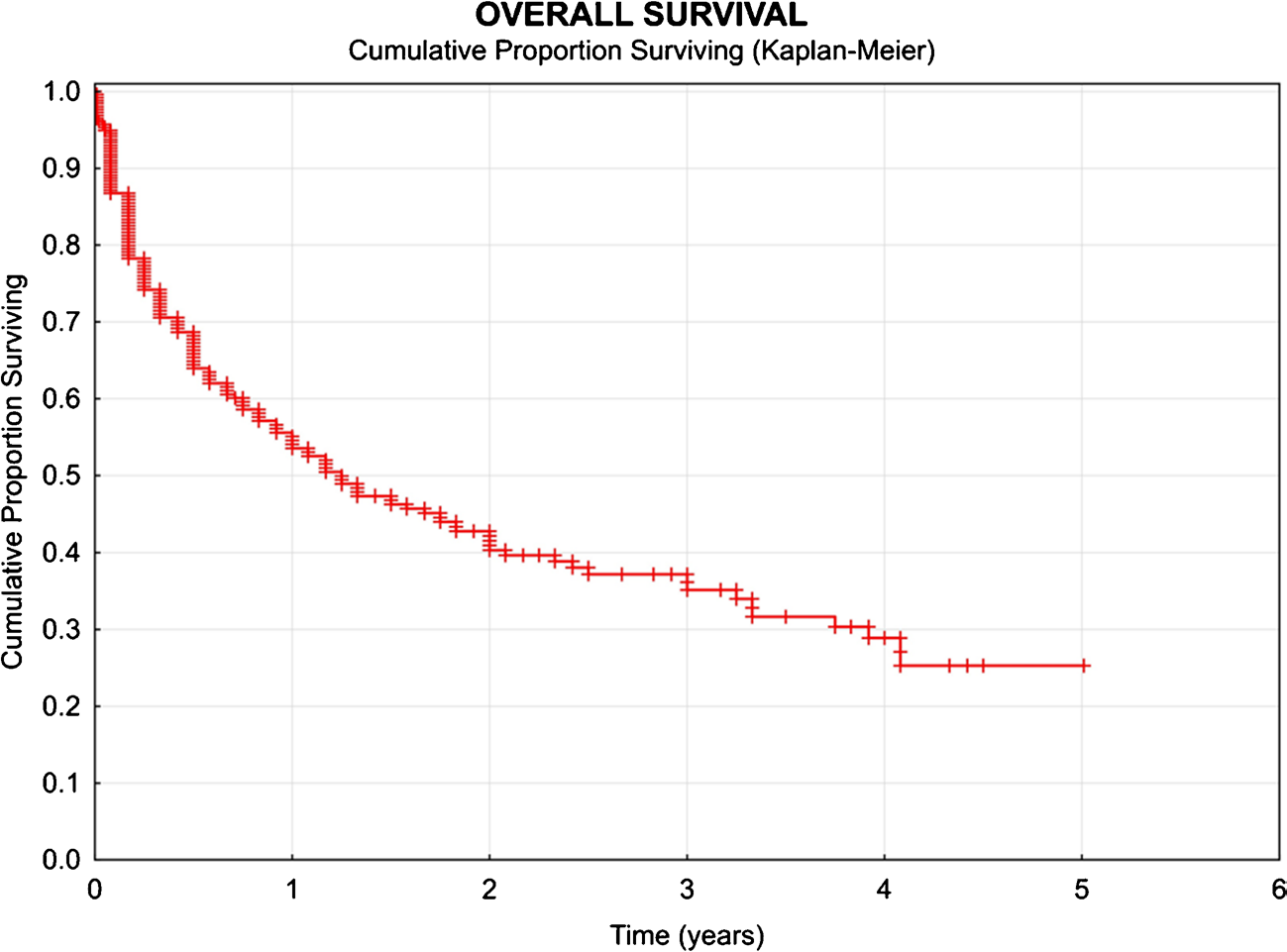
Overall survival of *KRAS* G12C adenocarcinomas by Kaplan–Meier univariate analysis showing poor 5-year OS with only 25% survival rate

Overall survival (OS) by variables and hazard ratios is shown in Table 4**.** Average survival was 1.89 years (SD 0.12); 1-year OS was 54%, dropping to 25% at 5 years **(**Fig. 4**)**.

**Table 4:** Statistical parameters in a relationship to 1-, 3- and 5-year overall survival (univariate analysis)

Variable	Subgroups		No of patients	1-year OS (%)	3-year OS	5-year OS (%)	Mean (yearsS)	SE	CI
OS			257	54	35	25	1.895	0.116	0.92–1.83
Age (p 0.0068)	> 73 years		65	45	22	17	1.267	0.164	0.50–1.25
	< 73 years		192	56	40	29	2.068	0.138	0.92–3.00
Sex (*p* = 0.0697	Male		142	49	29	20	1.623	0.137	0.71–1.75
	Female		115	59	42	32	2.145	0.182	0.75–4.08
Stage (*p* < 0.0001)	IA		22	89	68	NA	1.834	0.101	1.83-NA
	IB		11	100	90	72	3.100	0.201	1.75-NA
	IIA		3	100	100	100	NA	NA	NA
	IIB		13	83	65	64	1.114	0.102	0.92-NA
	IIIA		25	55	35	24	1.941	0.368	0.42–3.92
	IIIB		22	70	0	0	1.393	0.226	0.58–2.00
	IIIC		4	25	NA	NA	0.648	0.223	0.17-NA
	IVA		79	53	41	20	2.033	0.211	0.58–3.33
	IVB		51	22	10	NA	0.639	0.103	0.17–0.58
Therapy	Surgery *p* < 0.0001	No	197	47	25	21	1.505	0.116	0.50–1.25
		Yes	49	78	70	46	3.0616	0.229	3.25-NA
	RT *p* = 0.0135	No	164	48	32	22	1.713	0.143	0.50–1.33
		Yes	82	66	43	31	2.285	0.2002	1.25–4.08
	CHT *p* = 0.0322	No	135	47	35	29	1.644	0.154	0.33–1.75
		Yes	111	62	36	24	2.117	0.163	1.17–2.50
	IT *p* = 0.0077	No	190	48	33	23	1.746	0.137	0.50–1.33
		Yes	56	72	45	39	2.139	0.171	1.50-NA
Smoking history	Smokers		150	54	67	24	1.906	0.149	0.83–2.00
	Ex-smokers		74	53	32	32	1.568	0.153	0.58–2.42
	Non-smokers		5	30	30	NA	0.584	0.225	0.08-NA

*CHT* chemotherapy, *CI* confidence interval, *NA* not available, *IT* immune therapy, *OS* overall survival, *RT* radiotherapy, *SE* standard error

**Fig. 5:**
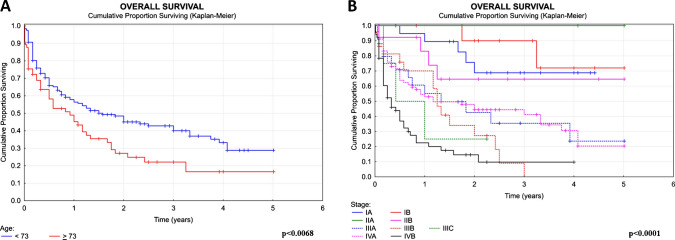
Overall survival of *KRAS* G12C adenocarcinomas was associated with patient age, with 73 years identified as the cut-off for the worst outcome (*p* = 0.0068) **(A)**, and with tumor stage, where higher stage correlated with poorer outcomes (*p* < 0.0001) **(B)**

Age > 73 years predicted worse outcome (HR 1.606, *p* = 0.0068) **(**Fig. 5A**)**. Gender was not statistically significant (*p* = 0.0697), though males had a lower 1-year (49%) and 5-year (20%) OS versus female patients (59% and 32%); males had a 1.353 times greater risk of dying from disease. Higher AJCC stage strongly correlated with poor OS (HR 3.03, *p* < 0.0001), with critical cut-off between stages IIB and IIIA (HR 5.09, *p* < 0.0001) (Fig. 5B**).** Type of therapy significantly influenced OS; absence of surgery (HR 2.987, *p* < 0.0001), radiotherapy (HR 1.57, *p* = 0.0135), chemotherapy (HR 1.43, *p* = 0.0322), and immunotherapy (HR 1.73, *p* = 0.0077) was each associated with poorer OS.

**Fig. 6:**
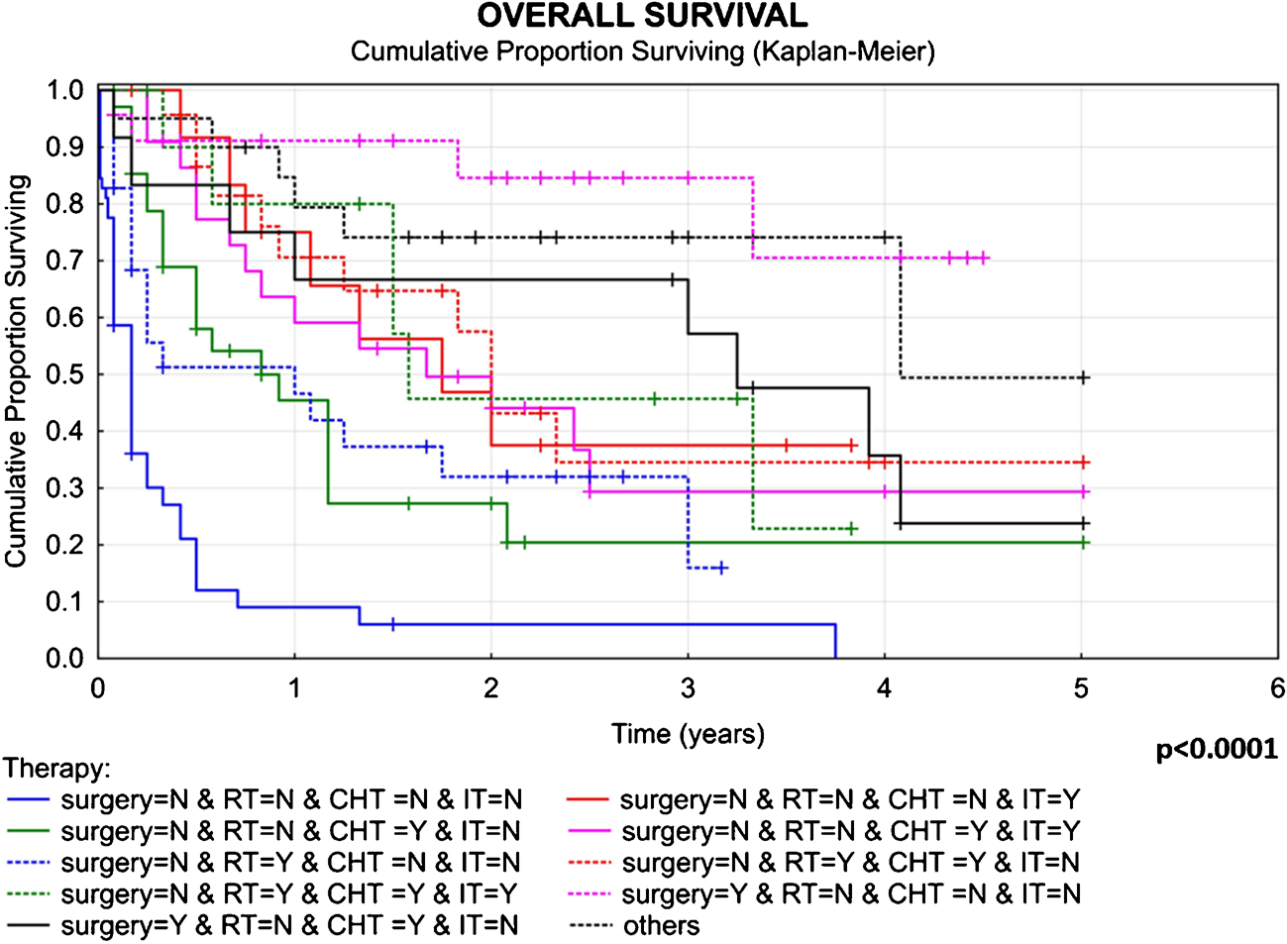
Overall survival of *KRAS* G12C adenocarcinomas by Kaplan–Meier univariate analysis related to different combinations of therapies

Analysis of different therapy combinations with at least 10 patients is presented in Fig. 6. As expected, patients who received no treatment (typically diagnosed at an advanced stage) had the poorest 1- and 5-year OS rates (9% and 0%, respectively, HR 5.998, *p* < 0.0001). Univariate analysis showed that the most statistically significant combination was surgery without radiotherapy, chemotherapy, or immunotherapy with *p* < 0.0001 and 1-year and 3-year survival rates 91% and 85%, respectively (the 5-year OS could not be calculated due to limited follow-up data) (Supplementary file 1). The HR for poor clinical outcome in this group was 0.184. Smoking had no statistically significant impact on OS; non-smokers (vs smokers and ex-smokers) showed worse OS with HR 0.730 (*p* = 0.5814, CI 0.730–0.232).

Multivariate analysis identified two factors significantly associated with OS: the number of therapies and disease stage. Patients who received one or more therapies and had a lower disease stage demonstrated longer OS. According to the Cox proportional hazards model (stepwise regression), the factors with the strongest negative association with OS were as follows (Supplementary file 2): no therapy (HR 7.204, CI 4.557–11.389), chemotherapy only (HR 2.044, CI 1.201–3.477), radiotherapy only (HR 3.192, CI 1.822–5.594), AJCC stage > IIB (HR 5.320, CI 2.820–10.038), and age over 73 years (HR 1.622, CI 1.095–2.402) (Supplementary File 3 – Regression tree).

## Discussion

*Kirsten rat sarcoma viral oncogene homolog (KRAS),* a principal isoform of the *RAS* gene, is the most common oncogenic driver in NSCLC [19]. About 80% of all *KRAS* oncogenic mutations occur at codon 12, with the most comomn variants being *p.G12D* (41%), *p.G12V* (28%), and *p.G12C* (14%) [7]. Among these, the *KRAS p.G12C* mutation is the predominant variant in NSCLC, accounting for 45–50% of all *KRAS* mutations and occurring in 11–16% of lung adenocarcinomas [8]. KRAS protein regulates proliferation by alternating between two stages: an inactive guanosine diphosphate (GDP)-bound form and an active guanosine triphosphate (GTP)-bound form. The *p.G12C* mutation impairs normal cycling activity between these two stages, by interfering with the binding of GTPase-activating proteins, hindering KRAS inactivation and resulting to accumulation of the pro-proliferative form [20].

The identification of tumor-driver gene mutations and/or fusions, along with the development of specific inhibitors, is essential for advanced treatment strategies and clinical outcomes. While EGFR- and TRK-inhibitors showed positive results in tumor-specific therapy [21, 22], RAS was, until recently, considered undruggable, due to two major challenges. First, KRAS has a small pocket on an otherwise smooth surface, making it unable to bind anything other than GTP. Second, GTP is abundant in normal cells, and the KRAS-GTP interaction is exceptionally strong, even at low GTP concentrations, because of its high binding affinity [5, 6]. On May 28, 2021, the Food and Drug Administration (FDA) approved AMG-210 (sotorasib) [11] and on December 12, 2022 MRTX849 (adagrasib) [12] as RAS GTPase family inhibitors for adult patients with *KRAS G12C*-mutated, locally advanced or metastatic NSCLC. Sotorasib was approved based on CodeBreaK 100 trial (NCT03600883) that involved 124 patients with disease progression after at least one prior systemic therapy [11, 20], The objective response rate (ORR) was 36%, with a median response duration of 10 months. Approval of Adagrasib was based on KRYSTAL-1 clinical trial (NCT03785249), which evaluated efficacy in 112 patients whose disease progressed after platinum-based chemotherapy and immune checkpoint inhibitor therapy [23, 24]. The ORR was 43%, with a median response duration of 8.5 months [12, 23].

In the present study, we retrospectively analyzed and reported baseline clinicopathologic characteristics of *KRAS* G12C-mutated NSCLCs that were not treated with RAS inhibitors. These tumors frequently exhibited solid growth, with squamous cell carcinoma (SCC)-like or rhabdoid/sarcomatoid phenotypes, while classic adenocarcinoma morphology was observed in only 28%, usually as a minor component. Most tumors expressed adenocarcinoma markers (TTF1, Napsin A), but some lacked these and showed only CK7 positivity; occasional p40/p63 and CK5/6 expression further complicated classification. Importantly, this overlap of morphology and immunoprofile, combined with SCC-like patterns, may lead to diagnostic inaccuracies and missed molecular testing.

PD-L1 expression was high across the cohort, with 22.2% of tumors showing positivity in 1–49% of tumor cells and 42.7% showing positivity in ≥ 50% of tumor cells; 35.0% were PD-L1-negative, this indicates that a substantional proportion of *KRAS* G12C-mutated NSCLCs may be candidates for immunotherapy, in addition to RAS-targeted therapy.

Co-mutations occur in a notable subset of *KRAS* G12C-mutated tumors, including *TP53*, *STK11*, *IDH1/2*, *PIK3CA*, *CTNNB1*, *MET*, *BRAF*, *FGFR2*, *FGFR3*, and *GNAS*. The presence of these additional genetic alterations expands the spectrum of potential therapeutic targets. For example, lorlatinib is the first-line therapy for ALK rearranged tumors [25, 26], tepotinib may be considered for tumors harboring *MET* amplifications or mutations [27]. A comprehensive molecular characterization also facilitates access to emerging therapeutic strategies that are currently investigational. These include gene therapy approaches, small molecules aimed at restoring tumor suppressor function (e.g., p53), and novel immunotherapeutic modalities. Several targeted agents under evaluation or approved for other indications may have relevance in this context: anlotinib, an antiangiogenic agent, for *TP53*-mutated NSCLC [28], ivosidenib (AG-120) for *IDH1*-mutated tumors, enasidenib (AG-221) for *IDH2*-mutated tumors [29], or alpelisib, a PI3Kα inhibitor, which has demonstrated efficacy in *PIK3CA*-mutated metastatic breast cancer and shows promise in other malignancies with PI3K pathway alterations [30, 31].

PD-L1 expression was high throughout the cohort with 52 cases setting in the expression of 1–49% and 100 cases with the expression over or equal to 50% of TPS score. Eighty cases were PD-L1 negative. When calculated across the entire tested cohort (n = 234, including negative cases), the mean PD-L1 expression was 39%. However, when considering only PD-L1-positive cases (n = 152, excluding negative cases), the mean expression was 60.6%. Such findings highlight the possibilities of the potential therapeutic target in a form of ICIs (pembrolizumab, nivolumab, etc.) in a single or combined therapeutic approach [32]. In lung adenocarcinomas, PD-L1 expression is significantly more frequent in poorly differentiated subtypes (solid and micropapillary) compared with well-differentiated ones [33, 34].

Clinically, *KRAS* G12C-mutated NSCLC represents aggressive malignancy with mean survival of 1.89 months (SD 0.12); with 1-year OS 54%, and a decline to 25% at 5 years. Poor outcome was associated with age > 73 years, AJCC stage > IIB, whereas combined therapy is associated with improved OS. Within our cohort, *TP53* co-mutations (*n* = 27) and *STK11* co-mutations (*n* = 12) were present but did not reach statistical significance as independent prognostic subgroups; notably, the TP53-co-mutated subset had 11 alive with disease at an average follow-up of 29 months and 14 disease-related deaths at 8.7 months, while the STK11-co-mutated subset included 10 disease-related deaths at 10.3 months and 2 survivors without evidence of disease. Although the overall KRAS G12C target remains clinically important, these data do not support the conclusion that KRAS G12C tumors are intrinsically more aggressive than other driver-defined tumors. Head-to-head comparisons in adequately powered cohorts, with standardized mutational panels and uniform treatment exposure, are required to define relative prognosis and to optimize genotype-guided therapy.

We identified another *FGFR3*::*TACC3* adenoid carcinoma diagnosed on a core-cut biopsy. The sample was largely necrotic, containing only a few viable tubular and cribriform areas, with no obvious clear cell morphology evident in the biopsy material. The entire tumor could not be assessed, but the patient was staged as cT4 N2 M1b with multiple lung and brain infiltrations; no additional FDG-avid sites were seen on PET/CT, suggesting a primary lung origin. The patient died of disease two months after diagnosis.

The second case, featuring an *LRP12*::*NRG1* gene fusion, showed solid growth with epithelial-to-sarcomatoid tumor cells and SCC-like features on core biopsy, staged cT3 N1 M1b, with death from disease at eight months. In both cases, napsin A and TTF-1 positivity supported a lung primary, and a full resection specimen was not available, autopsy was not indicated.

## Conclusion

*KRAS* G12C–mutated NSCLCs frequently exhibit solid morphology with SCC–like features, which may lead to misclassification and ommitment of proper molecular testing and, consequently, deprive patients of targeted treatment options beyond traditional chemotherapy and radiotherapy. Notably, *KRAS* G12C–mutated NSCLCs demonstrate a higher prevalence of PD-L1–positivity compared with non–*KRAS* G12C tumors, suggesting that immune checkpoint inhibitors could represent a more broadely applicable therapeutic option in this subgroup. Comprehensive molecular testing (e.g. NGS) further broadens treatment possibilities for this clinically aggressive tumor type.

## Supplementary Information

Below is the link to the electronic supplementary material.

ESM 1 (PNG 110 KB)

High resolution image (TIF 4.41 MB)

ESM 2 (PNG 193 KB)

High resolution image (TIF 8.47 MB)

ESM 3 (PNG 66.9 KB)

High resolution image (TIF 1.33 MB)

## Data Availability

Data supporting the findings of this study are available within the article. The complete datasets generated during and/or analyzed during the current study are available from the corresponding author upon reasonable request.
