# Supplementary figures and images for: Clinicopathologic features of KRAS G12C-mutated non-small cell lung carcinomas:insights from 279 retrospective cases
Source: Virchows Arch. 2026 Apr 15;489(1):49–63. doi: 10.1007/s00428-026-04526-x (PMC13369689; doi:10.1007/s00428-026-04526-x)

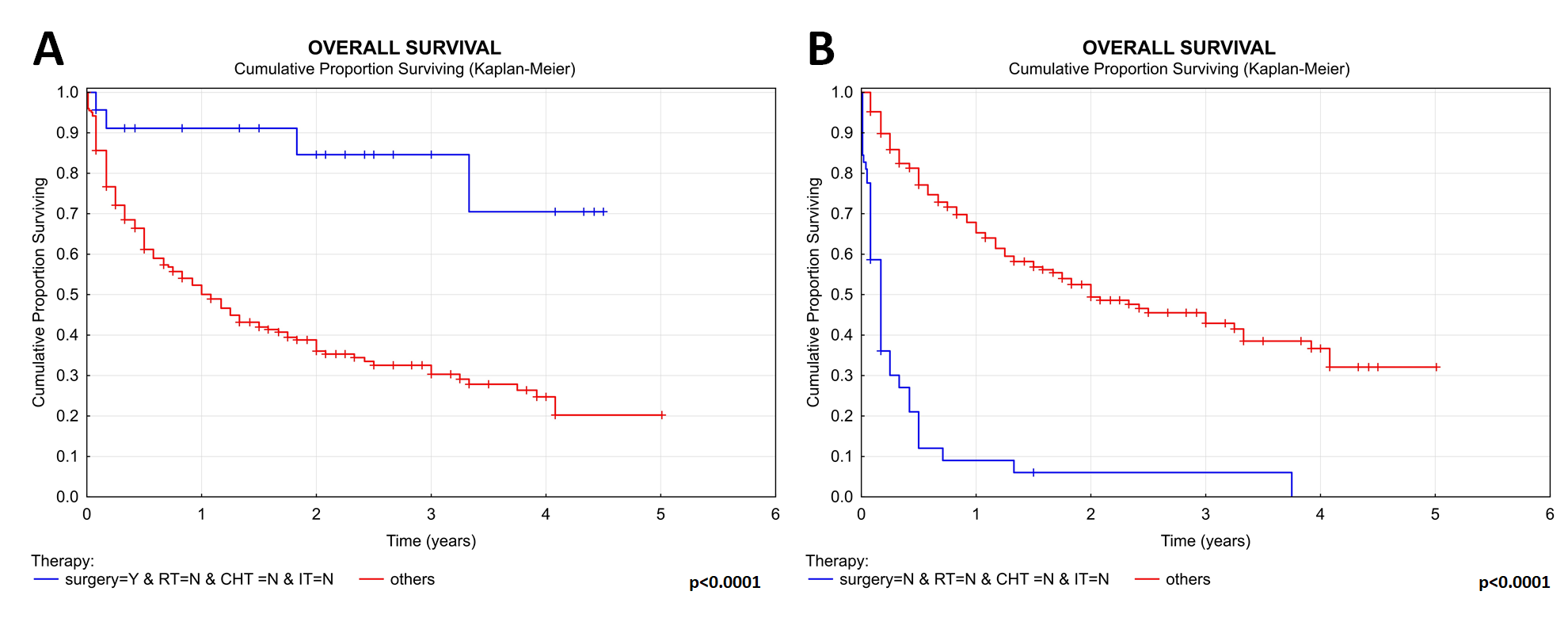

Supplement: Supplementary file 1 — (PNG 110 KB) [file 428_2026_4526_Fig7_ESM.png]

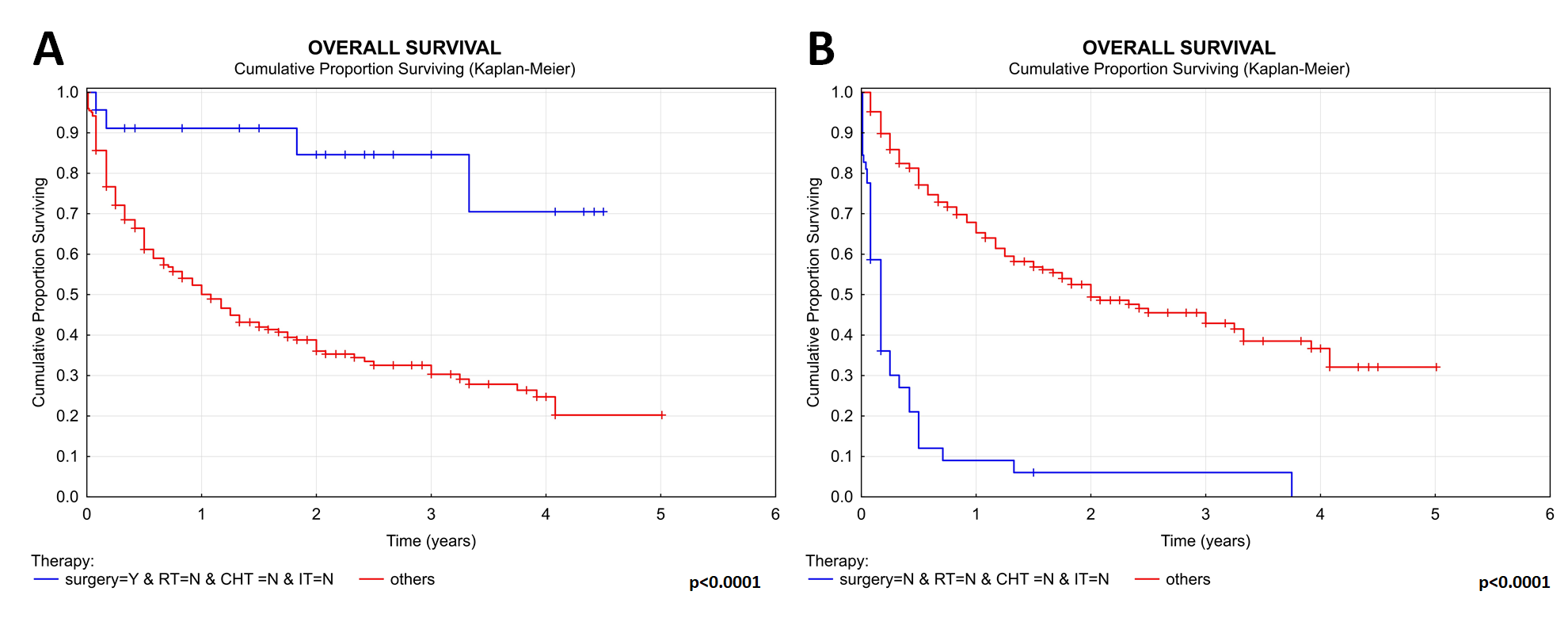

Supplement: Supplementary file 2 — High resolution image (TIF 4.41 MB) [file 428_2026_4526_MOESM1_ESM.tif]

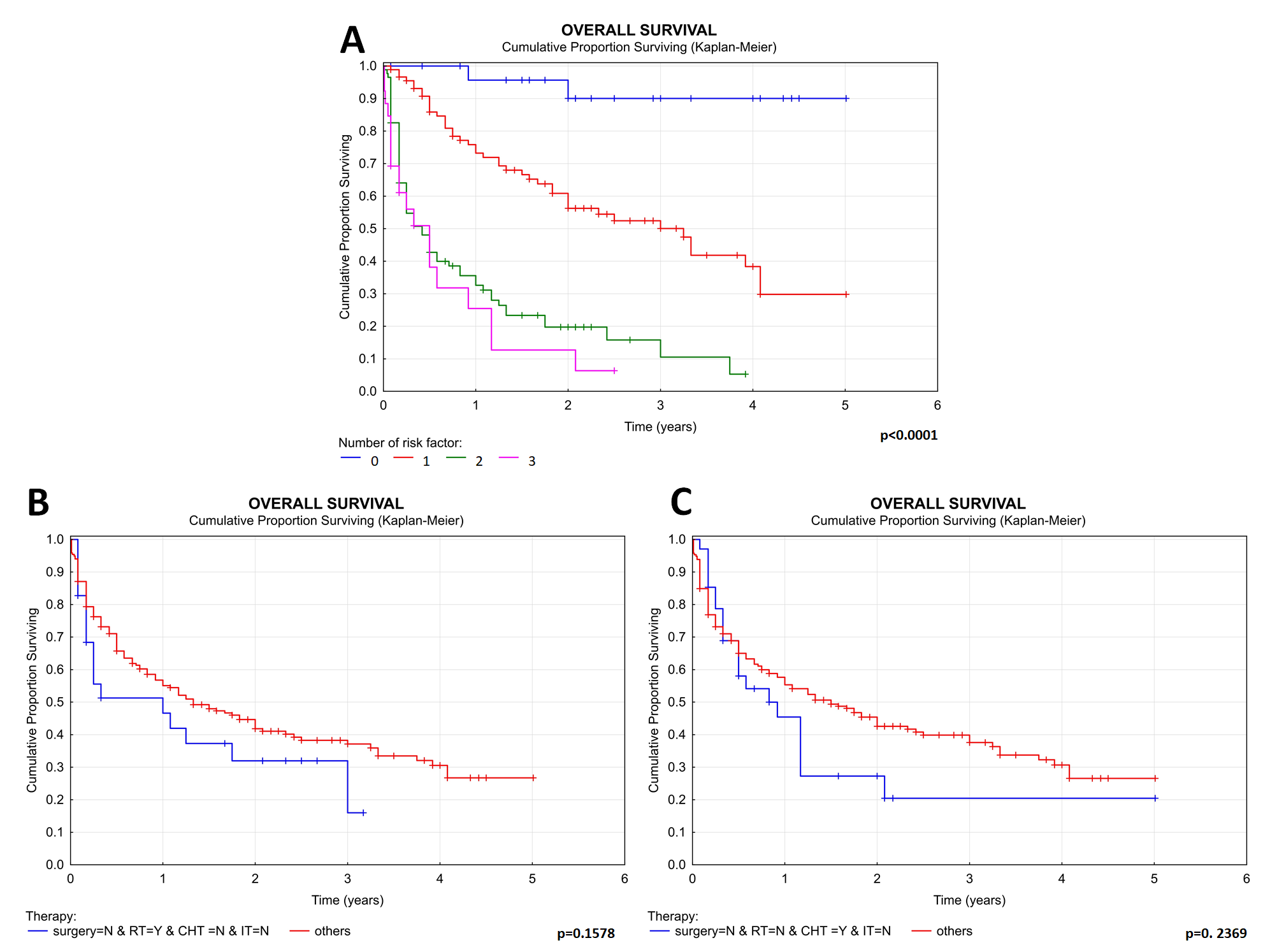

Supplement: Supplementary file 3 — (PNG 193 KB) [file 428_2026_4526_Fig8_ESM.png]

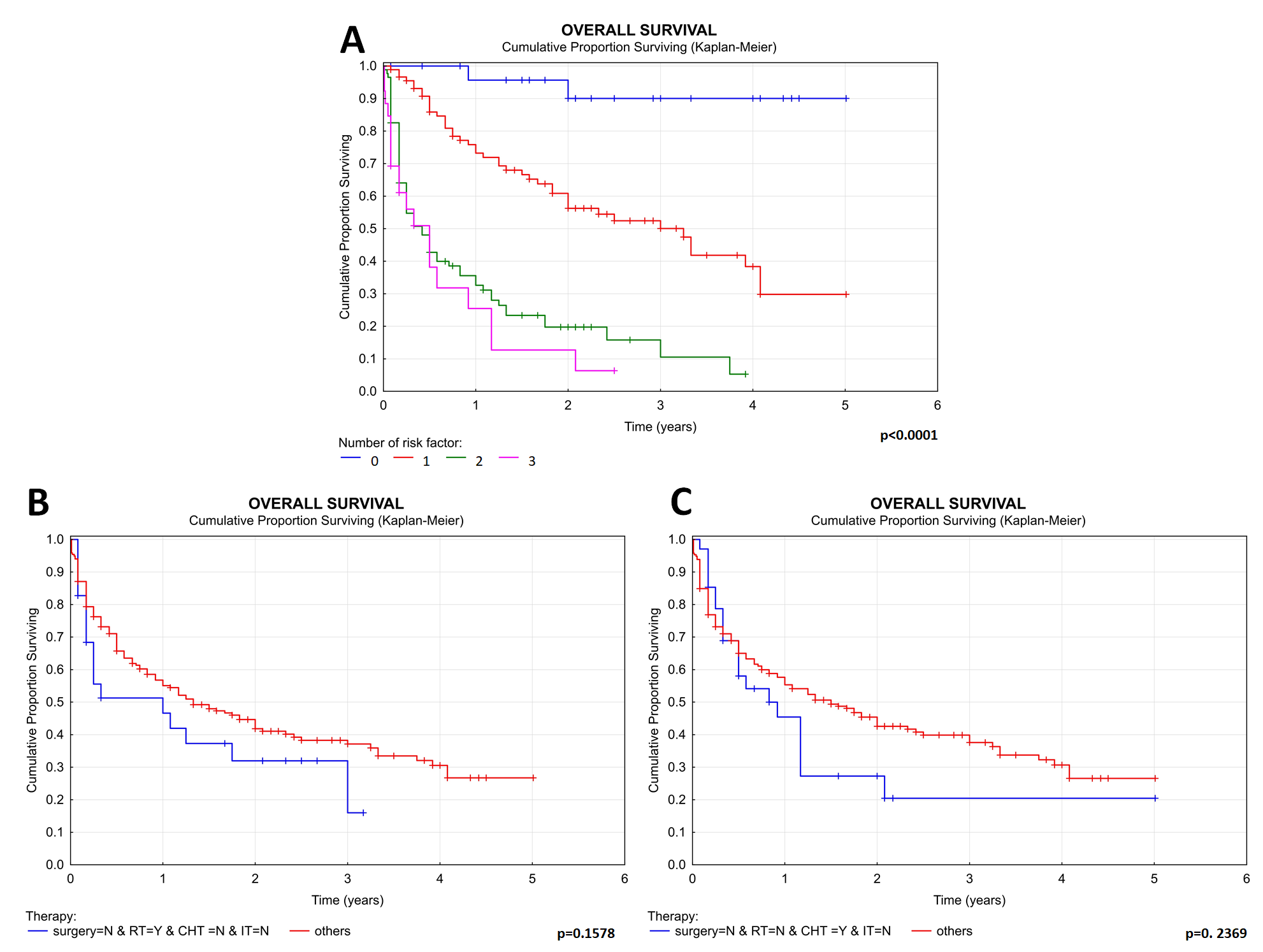

Supplement: Supplementary file 4 — High resolution image (TIF 8.47 MB) [file 428_2026_4526_MOESM2_ESM.tif]

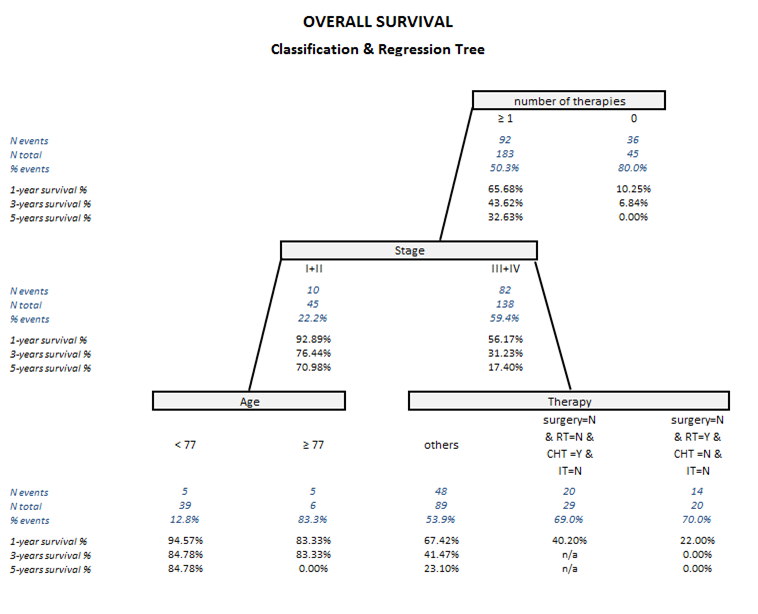

Supplement: Supplementary file 5 — (PNG 66.9 KB) [file 428_2026_4526_Fig9_ESM.png]

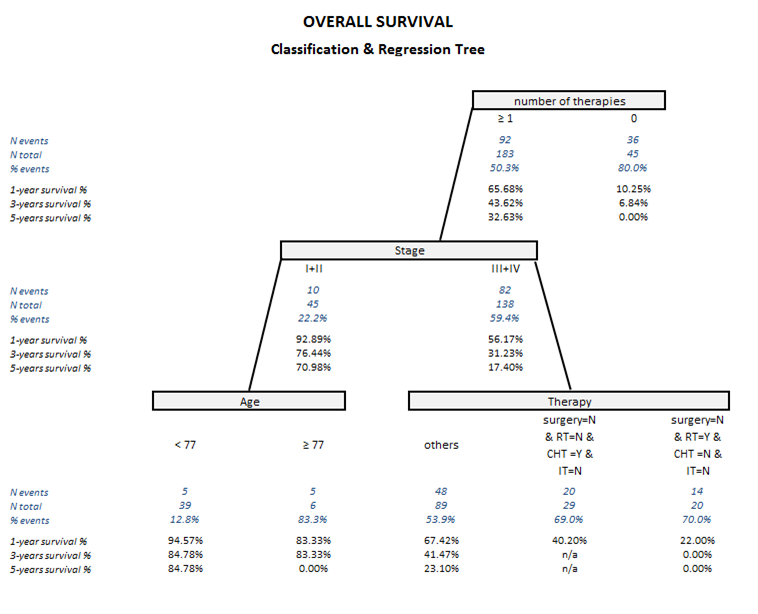

Supplement: Supplementary file 6 — High resolution image (TIF 1.33 MB) [file 428_2026_4526_MOESM3_ESM.tif]
